# Editorial: ChatGPT and other generative AI tools

**DOI:** 10.3389/fpsyg.2025.1535128

**Published:** 2025-02-05

**Authors:** Stefan Küchemann, Martina Rau, Knut Neumann, Jochen Kuhn

**Affiliations:** ^1^Chair of Physics Education Research, Faculty of Physics, Ludwig-Maximilians-Universität München (LMU Munich), Munich, Germany; ^2^Chair of Research on Learning and Instruction, Department of Humanities, Social and Political Sciences, ETH Zurich, Zurich, Switzerland; ^3^Department of Physics Education, IPN-Leibniz-Institute for Science and Mathematics Education, Kiel, Germany

**Keywords:** ChatGPT, personalized learning, large language model, learning with AI, critical thinking with AI, adaptive learning

## 1 Introduction

In the past ten years, applications of generative artificial intelligence (GAI) have found rapidly growing use in medicine, science, and the daily life. Large language models (LLMs) opened up new avenues in particular for education. LLMs have been used to create interactive educational content for students, stimulate their curiosity, generate code explanations, and develop assessment questions (Küchemann et al., 2023). However, there are also several challenges when integrating GAI in education.

This Research Topic aimed to address issues around the use of GAI tools to advance students' cognition or, more broadly, competencies, and how to enable both teachers and students to critically reflect upon the use of GAI tools instead of overly relying on them.

The Research Topic focused on research on the meaningful use of large language model-based GAI tools such as ChatGPT for learning and cognition in order to foster critical reflection in the field on how GAI tools can be used to support teachers in formative assessment, diagnosing students' difficulties, implement novel cognitive activities and targeted interventions, and provide individualized attention to students.

This editorial synthesizes insights from 14 studies in this Research Topic that investigate the diverse impact of AI in higher education, highlighting key themes in acceptance, assessment, performance comparison, skill development, interaction strategies, and cognitive modeling.

## 2 Relevant student characteristics related to GAI use in education

The following studies indicate, that students' acceptance and student-centered integration of GAI tools in education are critical for leveraging their potential benefits. For instance, Zou and Huang reveal a high intention to use ChatGPT among doctoral students for academic writing. Utilizing the Technology Acceptance Model (TAM), they find that students' attitudes significantly predict their intention to use AI, mediated by perceived usefulness and ease of use. Past experiences with ChatGPT enhance perceived ease of use, underscoring the importance of familiarity with AI tools.

Expanding on the role of acceptance, Yu et al. examine factors influencing user satisfaction and continued use of ChatGPT among college students. Their findings indicate that compatibility and efficiency positively affect perceived ease of use and usefulness, which in turn influence satisfaction and the intention to continue using AI tools. These studies collectively suggest that positive experiences and perceived benefits are crucial for integrating AI into educational practices.

Furthermore, Liang et al. explore the relationship between student interaction with generative AI and learning achievement. Through a survey of 389 participants, they find that interaction with AI tools positively correlates with learning outcomes, mediated by increases in self-efficacy and cognitive engagement. This implies that GAI tools can enhance learning by stimulating students' confidence and active participation in the learning process.

For a reliable assessment of self-efficacy in GAI usage among university students, Morales-García et al. adapt the General Self-Efficacy Scale. The resulting GSE-6AI scale is validated and found to be both reliable and invariant across genders, providing a valuable instrument for assessing students' self-efficacy related to GAI in educational settings.

The implementation of GAI in education settings necessitates the development of new skills among learners and educators. In this line, Federiakin et al. introduce Prompt Engineering as a critical 21st-century skill. Defined as the ability to articulate problems, context, and constraints to an AI assistant effectively, Prompt Engineering ensures accurate and swift AI responses. The authors propose a conceptual framework encompassing comprehension of prompt structure, prompt literacy, prompting methods, and critical online reasoning. Recognizing and cultivating these skills is essential for maximizing the benefits of AI tools in education and beyond.

Apart from that, Thüs et al. demonstrate how GAI can stimulate learning processes. In their article, they introduce OwlMentor, a GAI-powered learning environment designed to assist students in comprehending scientific texts. By integrating features like document-based chats and automatic question generation, OwlMentor aims to enhance student engagement with scientific literature. The results indicate that higher learning gains among users of OwlMentor, emphasizing the importance of aligning GAI tools with students' learning strategies to maximize learning outcomes.

## 3 Transforming assessment and scoring

The following articles demonstrate that the implementation of GAI into assessment practices presents both opportunities and challenges. Hackl et al. evaluate GPT-4's reliability as a rater for student responses in macroeconomics tasks. Their analysis reveals high inter-rater reliability, with Intraclass Correlation Coefficients ranging from 0.94 to 0.99, indicating that GPT-4 can produce consistent and reliable ratings. This suggests that AI could play an important role in standardized assessments, reducing the burden on human evaluators.

However, Kaldaras et al. caution against uncritical adoption of GAI in assessments. They highlight the challenges of ensuring that AI algorithms score the same constructs as human scorers and propose methods for evaluating the validity of GAI-generated assessments. Their work underscores the necessity of developing guidelines and methodologies to assess the validity of AI-based assessments and the inferences drawn from them.

Comparing AI with traditional methods, Kieser et al. find that conventional machine learning algorithms outperform a large language model in assessing students' concept use in physics problem-solving. This suggests that, in certain contexts, conventional AI algorithms may offer more accurate or efficient solutions than state-of-the-art GAI models, highlighting the importance of choosing appropriate AI tools for specific educational tasks.

Moreover, Küchemann et al. investigate the reliability and validity of concept inventory items generated by ChatGPT. After careful prompt engineering and selection, they create a set of physics concept questions that, while slightly lower in quality than human-generated items, are still viable for educational use. The study emphasizes the need for human oversight in generating assessment materials with AI to ensure alignment with learning objectives and student difficulties.

## 4 Analyses of GAI outputs

The comparison of GAI-generated outputs with human performance provides insight into the capabilities and limitations of GAI. Howe et al. conducted a study where participants compare advice from ChatGPT and professional advice columnists on social dilemmas. Surprisingly, ChatGPT's advice is perceived as more balanced, empathetic, and helpful, even when answer length is controlled. Although most participants prefer human advisors, the inability to distinguish between GAI and human responses raises questions about GAI's role in providing support and guidance.

During problem-solving of physics problems, Wang et al. examine GPT-4's ability to solve physics problems. While the AI model successfully solves 62.5% of well-specified problems, its performance drops significantly to 8.3% on under-specified, real-world problems. The identified reasons for failure—such as inaccurate physical modeling and unreasonable assumptions—highlight the current limitations of AI in complex, real-world applications and the necessity for human expertise in guiding AI use.

## 5 The role of human-GAI interaction in decision-making

Effective human-AI interaction strategies can significantly impact user engagement and decision-making. Yamamoto proposes a novel chatbot strategy employing suggestive endings inspired by the cliffhanger narrative technique. By ending responses with hints rather than conclusions, the chatbot stimulates users' curiosity and encourages deeper engagement. An online study demonstrates that users interacting with the suggestive chatbot ask more questions and engage in more prolonged decision-making processes, highlighting the potential of strategic AI communication to foster critical thinking.

In this line, Malloy and Gonzalez explore the application of GAI to cognitive models of decision-making. By categorizing existing applications and conducting an ablation study, they demonstrate that integrating GAI models to create memory representations and predict participant actions enhances model performance. This work provides valuable guidelines for cognitive modeling in in human-AI collaboration frameworks, suggesting that AI can augment our understanding of human cognition and improve decision-making models.

## 6 Conclusions

The studies in this Research Topic highlight the impact that generative AI is having across various facets of higher education. From relevant students' characteristics, student engagement as well as enhancing learning outcomes to transforming assessment practices, GAI tools like ChatGPT are reshaping the educational landscape. However, the authors of the articles also point toward challenges, including ensuring the validity and reliability of GAI-generated content, addressing limitations in GAI problem-solving capabilities, and fostering critical engagement rather than overreliance on AI outputs. To this end, educators, researchers, and policymakers must navigate these complexities thoughtfully. Embracing GAI's potential requires not only integrating these tools into educational practices but also critically assessing their impact, limitations, and the skills needed to use them effectively. By aligning GAI tools with educational objectives and student needs, fostering essential skills like Prompt Engineering, and maintaining human oversight in critical areas, the educational community can harness the benefits of AI while mitigating its challenges.

## References

[B1] Küchemann S. Steinert S. Revenga N. Schweinberger M. Dinc Y. Avila K. E.. (2023). Can chatgpt support prospective teachers in physics task development? Phys. Rev. Phys. Educ. Res. 19:020128. 10.1103/PhysRevPhysEducRes.19.020128

